# Flexible Sensors Based on Conductive Polymer Composites

**DOI:** 10.3390/s24144664

**Published:** 2024-07-18

**Authors:** Dan Zhao, Weiwei Jia, Xiaona Feng, Huali Yang, Yali Xie, Jie Shang, Pengjun Wang, Yufeng Guo, Run-Wei Li

**Affiliations:** 1 College of Integrated Circuit Science and Engineering, Nanjing University of Posts and Telecommunications, Nanjing 210023, China; zhaodan@nimte.ac.cn; 2 CAS Key Laboratory of Magnetic Materials and Devices, Ningbo Institute of Materials Technology and Engineering, Chinese Academy of Sciences, Ningbo 315201, China; 3 College of Materials Science and Opto-Electronic Technology, University of Chinese Academy of Sciences, Beijing 100049, China; 4 College of Electrical and Electronic Engineering, Wenzhou University, Wenzhou 325035, China

**Keywords:** flexible electronics, conductive polymer composites, flexible sensors, conductive mechanism

## Abstract

Elastic polymer-based conductive composites (EPCCs) are of great potential in the field of flexible sensors due to the advantages of designable functionality and thermal and chemical stability. As one of the popular choices for sensor electrodes and sensitive materials, considerable progress in EPCCs used in sensors has been made in recent years. In this review, we introduce the types and the conductive mechanisms of EPCCs. Furthermore, the recent advances in the application of EPCCs to sensors are also summarized. This review will provide guidance for the design and optimization of EPCCs and offer more possibilities for the development and application of flexible sensors.

## 1. Introduction

Flexible sensors enabled by soft electronic materials are shown to be important for robotics, healthcare and consumer/portable electronics due to the properties of wearability and excellent conformal capability [1,2,3,4,5,6]. As an emerging and important research area, flexible sensors have aroused great interest in the subject of how to realize both long-term and stable performance at tensile states. With the rapid development of soft robots, intelligent medical treatment and the human–machine interface, enormous efforts have been devoted into the development and improvement of flexible functional materials [7].

Elastic polymer-based conductive composites (EPCCs) composed of highly elastic polymer matrixes and conductive materials can withstand large deformation and are widely used in the fields of energy storage [8,9], energy harvesting, electromagnetic protection [10] and, especially, flexible sensors [11,12]. Usually, EPCCs are used as electrodes or sensitive materials in sensors. When used as sensitive materials, EPCCs can convert stress, temperature, light and other signals into electrical signals, which are easy to be detected. Rapid advances in the design and manufacturing methods of flexible sensors based on various polymer-based conductive composites have greatly expanded the application of flexible electronics.

According to the structures and preparation methods, conductive polymers can be divided into intrinsically conductive polymers and composite conductive polymers. Intrinsically conductive polymers refer to conjugated polymers whose molecular structure itself is electrically conductive or polymers that can be doped to obtain electrical conductivity, such as polyacetylene, polyaniline, polypyrrole, polythiophene and polyfuran. Composite conductive polymers refer to the materials prepared by adding a variety of conductive fillers into polymer matrixes, and then, the composites materials with good electrical conductivity and mechanical properties are prepared by physical and chemical regulation [13,14,15]. Due to the complexity of the structure and the difficulty of preparation and purification, most intrinsically conductive polymers are still in laboratory. Composite conductive polymers have been widely used because they can be processed in a similar measure with polymer materials. In this review, the types and conductive mechanisms of EPCCs are introduced, and the applications of EPCCs in various sensors are summarized.

## 2. Types of EPCC

**Figure 1:**
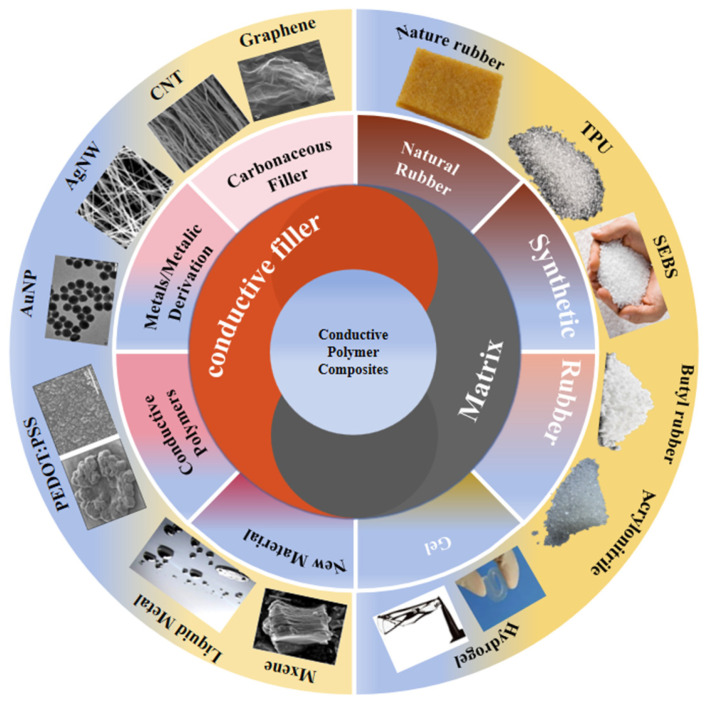
Material selection of EPCC. Reprinted with permission from Ref. [16]. Copyright © 2016 John Wiley and Sons. Reprinted with permission from Ref. [17]. Copyright © 2021, The American Association for the Advancement of Science.

The conductive properties and mechanical properties of EPCC are closely related to the types of polymer matrixes and conductive fillers. The popular polymer matrixes are elastomers such as natural rubber, synthetic rubber (thermoplastic polyurethane (TPU), Styrene Ethylene Butylene Styrene (SEBS), butyl rubber, acrylonitrile, etc.) and hydrogel, as shown in Figure 1. The selection of the matrix is mainly based on the application scenario. Rubber has excellent elasticity, electrical insulation and water resistance, but natural rubber easily ages and cannot withstand long-term use. Synthetic rubber is usually inexpensive and also has good elasticity. The production of synthetic rubber is not subject to regional restrictions. Synthetic rubber can be produced in large-scale in a short term. Properties such as acid and alkali resistance and the stability of physical properties at high and low temperatures are even better than natural rubber. Hydrogels are the most typical elastomers with three-dimensional (3D) network structure. They have the characteristics of sensitive response to stimulation, low surface friction coefficient, good flexibility and good biocompatibility. However, hydrogels are generally weak in mechanical properties and easy to break.

Traditional conductive fillers include carbon materials, metals and their compounds and intrinsically conductive polymers. Carbon materials are the most widely used conductive fillers. They are widely available, low-price and resistance-adjustable. However, carbon materials show poor dispersion in the preparation of composite materials. Moreover, the addition of carbon materials often reduces the mechanical properties of elastic materials. Metal and its compounds usually have excellent electrical conductivity and are easy to disperse in hybrid systems. Au nanoparticles (AuNPs) and Ag nanowire (AgNW) are most commonly used. However, most of the metallic materials used at present are expensive. The metallic materials will make the mechanical properties of the elastic matrix worse. Conductive polymers are mainly polyaniline, polypyrrole, polythiophene, etc. Intrinsically conductive polymers are generally unstable and poor in processability. They can achieve flexibility by combining with elastic polymers. The composites are more stable and easier to process. New conductive fillers such as Mxene, liquid metal and ionic liquid are also gradually being applied to the EPCC in the field of sensors with the development of material technology.

### 2.1. Polymer/Carbon Composites

**Figure 2:**
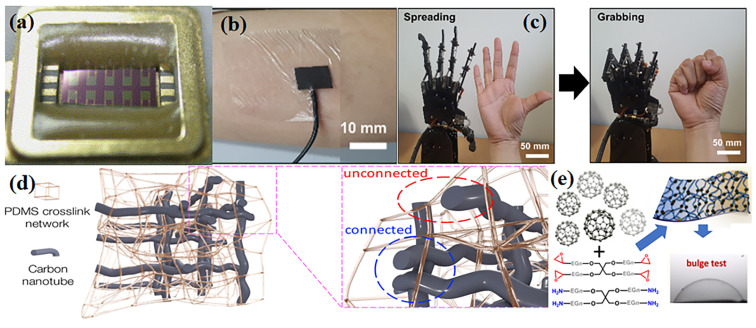
Polymer/carbon composites. (**a**) Sensing materials fabricated by mixing Poly (4-vinyl phenol), Poly (ethylene oxide), polycaprolactone with carbon black for gas sensor. Reprinted with permission from Ref. [24]. Copyright © 2005 Elsevier Ltd. (**b**) Self-healing polymer/graphene composite for a biosensor. (**c**) Self-healing polymer/graphene composite for interactive human–robot interface. Reprinted with permission from Ref. [19]. Copyright © 2022 MDPI. (**d**) Poly (dimethylsiloxane)/carbon nanotubes conductive composite. Copyright © 2018, American Chemical Society. (**e**) Poly (ethylene glycol)-fullerene composite for flexible sensors. Reprinted with permission from Ref. [32]. Copyright © 2023 American Chemical Society.

Polymer/carbon composites have been widely applied in electronic devices due to their mature preparation process and relatively low manufacturing cost. Carbon materials used at present are carbon black (CB), carbon nanotubes and graphene [18,19,20,21,22,23]. Carbon black is a kind of natural semiconductor with a volume resistivity of 0.1~10 Ω·cm. The volume resistivity of polymer materials can be greatly adjusted by selecting different addition amounts of CB. Xie et al. reported a gas sensor produced by depositing polymer/CB sensitive membranes on the microelectrodes. The sensor displayed a relative change in the ratios of resistance ΔR/R ranging from 3.2% to less than 0.1%, and the response time to the organic gas was 10 s [24], as shown in Figure 2a.

The great length–diameter ratio of carbon nanotubes (L/D > 1000) is beneficial to form a three-dimensional network structure of conductive channels. When the percolation threshold is reached, the amount of multi-walled carbon nanotubes is less than 1/10 of conductive CB. A small amount of carbon nanotubes endow the material with advantages such as better processability and better mechanical properties. Gan et al. used carbon nanotubes (CNTs) with diameter of 9.5 nm and length of 1.5 μm as nanofillers and polydimethylsiloxane (PDMS) as the matrix. The percolation threshold of nanofillers could be reduced to 0.06 wt%. By establishing a dual network model of crosslinking and conductive networks, the connection of one-dimensional nanofillers could be improved to stabilize the conductive networks [19], shown as Figure 2b.

Graphene, known as a two-dimensional material, can usually remain in contact to maintain the conductivity of the material when the composites are stretched. The surface of reduced graphene oxide (RGO), which is now widely used in sensors, contains a large number of functional groups that is beneficial to compounds with a variety of materials. Song et al. reported a self-healing polymer/graphene composite that can autonomously heal itself at room temperature. The composite had excellent electrical recoverability even from repeated scratches and remained stable after stretching [25].

In addition to the above carbon materials, carbon materials such as fullerenes and carbon quantum dots are also used in the field of sensors [26,27,28,29]. Shi et al. prepared three-dimensional nanocomposites using fullerenes, one-dimensional carbon nanotubes and two-dimensional MXene. The composite was used in flexible strain sensors. Due to the presence of fullerenes, the friction between adjacent layers of two-dimensional materials was reduced, and the sensing performance was improved (>400, >50% strain) [30]. Zhao et al. reported a new type of composite material prepared by using carbon quantum dots and nanofiber clusters as the sensitive material of the flexible humidity sensor. The sensor can be used for environmental humidity monitoring, plant transpiration monitoring, diaper humidity monitoring and so on [31].

### 2.2. Polymer/Metal Composites

Although polymer/carbon composites have been widely used, carbon materials are poorly dispersed and easily agglomerate when combined with polymers, increasing the difficulty of processing. Metal materials have better conductivity and dispersibility than carbon materials. All kinds of nano-metal particles and nanowires such as gold, silver, platinum nanoparticles, silver nanowires, copper nanowires, etc., have been used in polymer/metal composite conductive materials [33,34,35,36]. Jiang et al. combined gold with Styrene Ethylene Butylene Styrene (SEBS) to prepare a highly stretchable biphasic nano-dispersed (BIND) interface. This design realized the integration of soft, rigid and packaged modules and formed a stretchable hybrid device in a plug-and-play manner that can be applied to implantable human–computer interaction. Liquid metal (LM) has been regarded as an ideal conductive material in flexible electronics due to its good electrical conductivity and fluidity. There have been numerous reports on the synthesis of EPCC by using liquid metal/polymer composites [37,38,39,40]. Cao et al. reported a stretchable electrode based on liquid metal nanoparticles and a thermoplastic polyurethane (TPU) nanofiber. Without alloying or adding binder materials, the electrode showed good robustness and can be used for e-skins in human–machine interaction. The conductive pathway in the electrode can be reconstructed after damage [40].

### 2.3. Insulating/Conductive Polymer Composites

Generally speaking, polymers are usually insulators, but with the discovery of polyaniline, polythiophene, etc., intrinsically conductive polymers are gradually being applied in energy, optoelectronic devices, sensors and other aspects. However, most of the intrinsically conductive polymers have poor processability and are unstable in air. In addition, their flexibility cannot meet the application requirements of flexible electronics. The use of insulating/conductive polymers can overcome the problem of instability and improve their processability [41]. Wang et al. utilized PEDOT: PSS and poly (ethylene glycol) diglycidyl ether (PEGDE) to directly covalently crosslink, so that the stretchability and conductivity of PEDOT: PSS films were significantly improved. The crosslinked elastomer displayed a stretchability of 50% and a conductivity of 13,100 S·m^−1^ by optimizing the reaction temperature and PEGDE ratio [42].

### 2.4. Polymer/Mxene Composites

**Figure 3:**
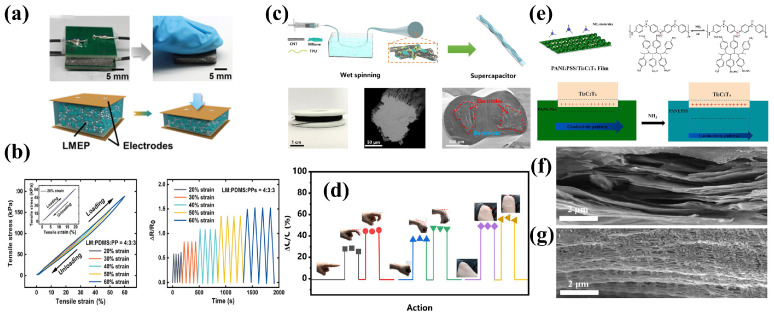
Polymer-based composites. (**a**) PDMS/LM composite elaster for capacitive stress sensors. (**b**) Characterization of the capacitive stress sensor based on PDMS/LM composite. Reprinted with permission from Ref. [38]. Copyright © 2022 John Wiley and Sons. (**c**) CNTs/MXene-TPU fiber for flexible strain sensors. (**d**) Strain sensors for action detecting. Reprinted with permission from Ref. [45]. Copyright © 2021 Elsevier Ltd. (**e**) PANI: PSS/Ti_3_C_2_T_x_ film for NH_3_ detecting. (**f**) Ti_3_C_2_T_x_ film and (**g**) PANI: PSS/Ti_3_C_2_T_x_ film. Reprinted with permission from Ref. [46]. Copyright © 2022 Elsevier B.V.

MXene consists of transition metal carbides, nitrides or carbon nitrides of several atomic layers’ thickness and has attracted wide attention as a two-dimensional material due to its high specific surface area and complex surface chemical behavior in electrical, optical, thermal, magnetic, mechanical and surface modifiability, etc. Since MXene materials have hydroxyl groups or terminal oxygen on their surfaces, they have the metallic conductivity of transition metal carbides and can almost meet the requirements of both the sensing and mechanical properties of flexible sensors at the same time. MXene has been used in various flexible sensors such as pressure sensors, strain sensors, temperature sensors, biochemical sensors and gas sensors. The hydrophilic surface and highly adjustable surface groups of MXene make it easier to form composites with polymer matrixes. The properties of polymer matrixes can be significantly improved so that their application range are broadened [43,44,45]. Wu et al. prepared a kind of stretchable conductive CNTs/MXene-TPU hybrid fibers through the wet-spinning method. The hybrid fibers can also be applied to strain sensors with high sensitivity, fast response and good flexibility [45], as shown in Figure 3c,d.

### 2.5. Polymer/Mixed Filler Composites

Conductive fillers with different sizes, structures and characteristics show better stability in the deformation process by constructing conductive networks, and the mixed fillers can also improve the mechanical properties of materials. Therefore, many researchers choose mixed fillers to improve the properties of materials [47,48,49]. Pimalai et al. proposed a novel electrochemical sensor for paraquat detection by combining GO-Au nano-particles and electropolymerized poly(3-aminobenzoic acid) modified electrode. The sensor had a wide paraquat detection range and good linear correlation, and the detection limit was as low as 0.45 nmol/L. Wen et al. developed flexible poly (4-stryenesulfonate)-doped polyaniline nanoparticle (PANI: PSS)/Ti_3_C_2_T_x_ composites to detect NH_3_ by in situ polymerization. The gas sensor based on the optimized film displayed the high response of 0.57–1 ppm NH_3_. Additionally, this gas sensor obtained excellent mechanical stability and a low detection limit (20 ppb) at room temperature [46], as shown in Figure 3e–g.

## 3. Conductive Mechanism of EPCC

The resistivity of the EPCC varies with the change in conductive filler and almost does not decrease with the increase in conductive fillers when the amount is low. However, when the amount of conductive fillers reaches a specific value, a tiny extra amount can reduce the resistivity of the material by several orders of magnitude. When the addition of conductive filler further increases beyond a specific value, the resistivity of the composites tends to be stable again, and further increase in filler can no longer improve the resistivity of the material significantly. The S-shaped curve relationship between the addition of conductive filler and the resistivity of the material is called the conductive percolation, and the value of the point is the conductive percolation threshold.

Many theoretical models are proposed based on the basic principles of the physical conductive path and chemical electron transition to explain the mechanism when the conductive fillers are in contact completely, in a small distance and in a certain distance. The mechanism can be classified into three categories, namely the corresponding conductive path theory, the electron tunnel effect theory and the field emission theory.

### 3.1. Conduction Path Theory

**Figure 4:**
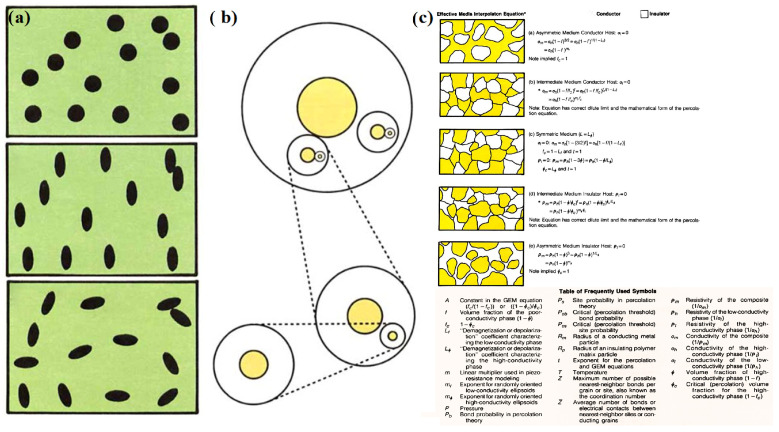
(**a**) Maxwell infinite dilution model for spheres in a conducting matrix. (**b**) Bruggeman efficient medium theory. (**c**) Effective media interpolation equation. Copyright © 2005 John Wiley and Sons. Reprinted with permission from Ref. [52].

The conductive path theory is the most traditional theory, which holds that the resistivity of the material is high when the amount of conductive fillers is low, because the fillers are isolated by polymer layers and cannot contact each other. When the amount of conductive fillers increases to a certain extent, fillers can come in contact to form a conductive network, and the resistivity of the material begins to decrease significantly because electron transmission channels in the composites are constructed. When the amount of conductive filler increases to a certain extent, the electron transport channels in the material tend to be saturated, and the corresponding resistivity no longer changes significantly [50,51]. Many empirical formulas and theoretical models have been established to prove the conduction path theory. In the 1990s, McLachlan [52] combined the Maxwell infinite dilution model [53] and Bruggeman’s effective medium theory [54], took the effects of the shape and dispersion of conductive media on conductivity into account and improved the model by proposing a universal equation for the effective medium, as shown in Figure 4. The above two kinds of theoretical models are widely accepted as the guidance of conductive path theory and play a guiding role in the study of force-sensitive flexible materials. However, the models are limited to the analysis of the geometric morphology of conductive fillers and the number of contacts between particles. The influence of the interface relationship between the polymer and conductive medium are not considered either; therefore, Wessling et al. proposed a dynamic interface model in 1991 based on certain assumptions [55], making the theory more perfect. The conductive path theory based on the physical conduction mechanism of current corresponds well to the macroscopic phenomenon of conductive percolation and the change in material microstructure and provides theoretical guidance for the microstructure optimization design of polymer-based conductive composites [56,57].

### 3.2. Electron Tunneling Theory

The polymer composites often display a change in resistivity before the amount of onductive fillers reaches the theoretical content required to build a conductive path. This phenomenon indicates that the conductive function can be realized when the conductive fillers in the polymer matrix are not completely in close contact, which also means that the conductive path theory is imperfect. The researchers further propose that when the space between the conductive fillers is small, the electrons in the outer atomic layer of the conductive fillers can cross the potential barrier after absorbing a certain amount of energy and transfer between the particles in incomplete contact, thus forming a tunnel current and realizing conduction. This is also known as the electron tunneling theory [58]. According to Simmons [59], the tunnel current density of composite conductive materials can be expressed as follows:

$$J=\frac{3e^{2}V\sqrt{2m\varphi }}{2\omega h^{2}}\times exp\left(-\frac{4\pi \sqrt{2m\varphi }}{h}\right)$$

where *J* is tunnel current density; *m* mass of the electron; *h* Planck’s constant; *V* voltage; *φ* thickness of insulation; *e* electron charge; *φ* barrier height.

The tunneling effect occurs only in the very small area where the conducting medium is close to each other. The tunnel current only exists when the conducting medium content reaches a certain concentration, and the concentration range is very small, so the tunnel current theory is greatly limited.

### 3.3. Field Emission Theory

**Figure 5:**
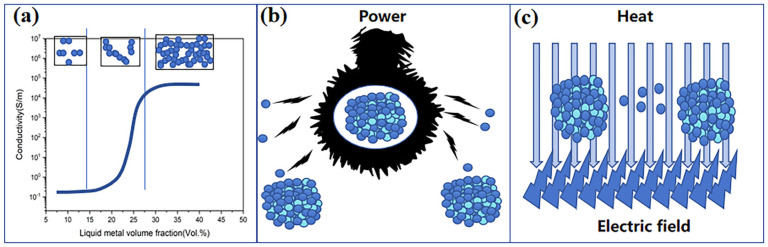
Schematic of conductive mechanism. (**a**) Conduction path theory. (**b**) Electron tunneling theory. (**c**) Field emission theory.

In addition to the above theories, it is also found that under the effect of higher external electric field or higher ambient temperature, the polymer composites can be conductive as well when the amount of conductive fillers is low. Analysis suggests that the conductive fillers can form a strong electric field between each other due to strong external action. In this way, electrons can be continuously emitted from one particle to another at a certain distance. The phenomenon that electrons can produce electric current through emission transition under strong action is called field emission theory [60,61]. The above theories successively reveal the conduction mechanism when the conductive fillers are in contact completely, in a small distance and in a certain distance, comprehensively explain the physical structure basis of the conductive percolation phenomenon and also explain the reasons for the current path construction under different conditions of polymer-based conductive materials. These theories provide theoretical guidance for the microstructure optimization of polymer composite conductive materials. As shown in Figure 5.

## 4. Flexible Sensors Based on Conductive Polymer Composites

Flexible electronic devices have been more and more widely used in health monitoring, motion capture, intelligent prosthetics and robots due to their mobility, lightweight, sustainability, simplicity and interactivity. As a key component of wearable electronic devices, the improvement of sensor performance is particularly important [62,63,64,65]. The existing classifications of sensors are complex and can be divided roughly into force sensors, mainly including pressure sensors and strain sensors, temperature sensors, biochemical sensors and humidity sensors according to different specific functions. Although the sensors work under different principles, they can be boiled down to using some physical, chemical or biological characters of the sensing materials to collect the required information and convert it into electrical signals. In order to obtain high-performance flexible sensors, it is necessary to select appropriate materials based on the requirements of sensitivity, hysteresis, detection range, service life and other considerations.

Sensors for different applications have different material requirements. Strain sensors require materials that can withstand large deformations, so more attention is paid to the tensile properties of the material. Temperature sensors require materials to have relatively stable physical and chemical properties over a wide temperature range, and the specific heat capacity should be as small as possible to avoid the impact of thermal inertia. For biosensors, bio-sensitive materials are required by different biochemical reactions. It is also necessary to select a carrier material that is both biocompatible and has a strong fixing effect on the sensitive materials to fix the substances with recognition function. The sensitive materials of gas sensors need to possess high selectivity and stability in order to identify the gas to be detected accurately among the complex interference factors.

### 4.1. Flexible Pressure Sensors

Flexible pressure sensors have greater advantages than rigid sensors in human–computer interaction, medical health, robotic haptics and other applications but also put more stringent requirements on materials. The material should be thin, soft, long-term reliable and, in some cases, able to be attached to human skin or implanted in the human body. Some materials used in flexible pressure sensors are shown in Table 1.

**Figure 6:**
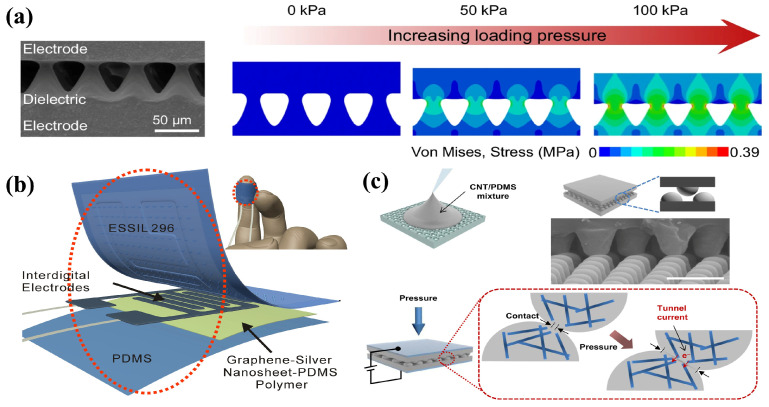
Polymer-based composite conductive materials are used for flexible pressure sensors. (**a**) Pressure sensor with a bonded interface and finite element simulations under the loading process. Reprinted with permission from Ref. [66]. Copyright © 2024 Springer Nature (**b**) Piezoresistive type flexible pressure sensor consists of interdigital electrodes and graphene-silver-nanosheet-polymer nanocomposites as a sensing layer. Reprinted with permission from Ref. [67]. Copyright © 2023 MDPI. (**c**) CNT/PDMS conductive composite elastomers with interlocked microdome arrays used as a flexible pressure sensor. Reprinted with permission from Ref. [68]. Copyright © 2014, American Chemical Society.

Flexible pressure sensors can be divided into piezoresistive, capacitive, piezoelectric and triboelectric sensors. Sensitivity, detection range, linearity, response time and cycle stability are the main parameters [71]. Zhang et al. studied the influence of energy dissipation at the microstructure interface on the recovery speed of a flexible piezocapacitive. They adopted the integrated bonding technology of the microstructure interface to extend the frequency bandwidth of the sensors from hundreds of Hertz to at least 12,500 Hz and also exhibited a high frequency resolution of 0.2 Hz at 1 kHz, which showed negligible capacitance-pressure hysteresis [66], as shown in Figure 6a. Ha et al. reported a flexible composite response pressure sensor prepared by doping carbon nanotubes into Ecoflex. The sensor exhibited a composite response of piezoelectric capacitance and piezoelectric resistance and displayed a significant increase in sensitivity (more than 400%) over a wide pressure range (3.13 kPa^−1^, 0.43 kPa^−1^, 30–50 kPa) [72]. Flexible pressure sensors also show great application potential in extreme conditions. Lin et al. reported a multi-wall carbon nanotube (MWCNT)/graphite (GP)/thermoplastic polyurethane (TPU) composite as the sensing layers of a pressure sensor. The sensor showed great robustness through material design with near-zero resistance temperature coefficient (TCR), radial gradient partial pressure microstructure and flexible interface bonding process. The sensor achieves a near-zero resistance temperature coefficient over a temperature range of 25–70 °C and is capable of achieving signal fluctuations as low as 0.6% and high interface strength of up to 1200 kPa [69].

### 4.2. Flexible Strain Sensors

**Figure 7:**
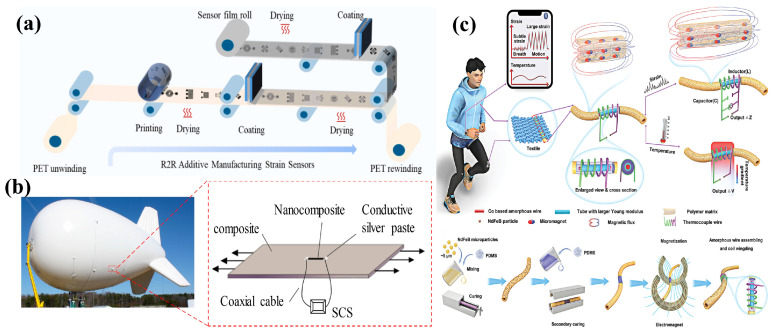
Polymer-based composite conductive materials are used for flexible strain sensors. (**a**) Schematic diagram of additive manufacturing sensor films using MWCNTs/PDMS composites. Reprinted with permission from Ref. [73]. Copyright © 2022 Elsevier Ltd. (**b**) Crack detection tests of aerostats with the CB/MWCNTs/PDMS nanocomposites strain sensor. Reprinted with permission from Ref. [74]. Copyright © 2017 MDPI. (**c**) NdFeB@PDMS magnetic composites used in a strain and temperature dual-mode sensor. Reprinted with permission from Ref. [77]. Copyright 2023 John Wiley and Sons.

The strain sensor converts the mechanical signals of material deformation into electrical signals, so the material should have a good deformation ability. Sensitivity and gauge factor, stretchability, linearity and reliability are the main parameters. Materials and parameters of flexible strain sensors are partly shown in Table 2. Yang et al. utilized 3-Methacryloxypropyltrimethoxysilane modified MWCNTs/PDMS conductive composites as flexible strain sensor films. The printed sensors based on the MWCNTs/PDMS composites exhibited a sensitivity of 1.55 when stretched to 100%. It can be used as a wearable electronic device for motion detection, sports and rehabilitation training and structural health monitoring [73], as shown in Figure 7a. In addition to health monitoring, flexible strain sensors can also be used for structural damage and crack monitoring, the sensors here are not required to have a large deformability but high sensitivity and durability. Yin et al. prepared high-durability ternary polymer conductive nanocomposites using polydimethylsiloxane (PDMS), carbon black nanoparticles and multi-wall carbon nanotubes (MWCNTs). They realized a stretchable strain sensor with excellent durability (more than 10^5^ cycles under 25% strain), high sensitivity (GF ~12.15) and good linearity and reproducibility. The sensors were used to detect cracks for the aerostats surface and showed great potential in structure health monitoring [74], as shown in Figure 7b.

**Table 2:** Materials and parameters of flexible strain sensors.

Materials	Production Methods	Detection Range	Sensitivity(GF)	Ref.
MWCNTs/PDMS	Screen printing	0~100% strain	1.55	[73]
CB/MWCNTs/PDMS	Screen printing	0~25% strain	~12.25	[74]
LM/PDMS	Evaporation	0~50% strain	--	[75]
GO-AgNW/PVA	Screen-printing	>50% strain	>100	[76]
NdFeB@PDMS	Mixing	0~20% strain	5.29	[77]

### 4.3. Flexible Temperature Sensors

Temperature is the most basic physical parameter that reflects the state of an object and environment. The strict control of temperature is of great significance in daily life and industrial production. With the rapid development of smart wearable devices and temperature monitoring systems, properties of temperature sensors such as temperature measurement range, sensitivity, response time and temperature resolution are expected to be improved to meet practical application requirements [78]. Some materials and parameters are shown in Table 3.

**Figure 8:**
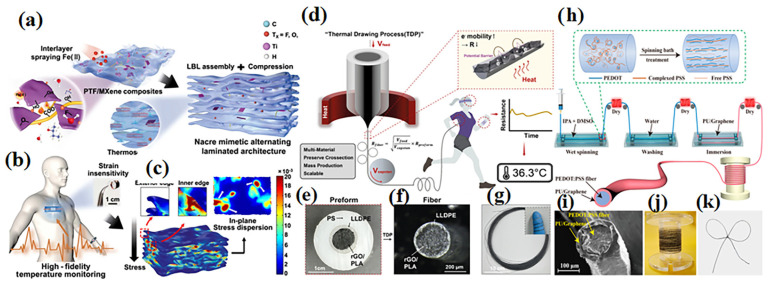
Flexible temperature sensor (**a**) PFT/Mxene/Fe composites are used as sensing layer. (**b**) Application of assembled temperature sensor for high-fidelity skin temperature monitoring. (**c**) The simulation of the composites’ structure. Reprinted with permission from Ref. [79]. Copyright © 2022 Springer Nature. (**d**) Fabrication of polymer-nanocomposite-fiber temperature sensor. (**e**) Cross-sectional photograph of the multi-layer fiber. (**f**) Cross-sectional optical microscopic image of the fiber temperature sensor after the thermal drawing and etching processes. (**g**) Photograph of a bundle of fiber temperature sensors before PS etching (inset image: fiber flexibility). Reprinted with permission from Ref. [82]. Copyright © 2023, The Springer Nature. (**h**) Preparation and characterization of PU/graphene encapsulated PEDOT: PSS fiber (composites fiber) with skin-core structure. (**i**) SEM image of the composites’ fiber cross-section. (**j**) Composite fibers packaged on the reel; (**k**) knotted composite fibers. Reprinted with permission from Ref. [87]. Copyright © 2023 American Chemical Society.

At present, polymer-based conductive composites used for temperature sensors mainly include polymer/metal composites, polymer/carbon composites, conductive polymer materials and the mixture. The most common sensitive materials are Pt, Au, metal oxide, carbide, etc. The high temperature resistivity of metal oxide materials can improve the temperature sensing performance. Inspired by the nacre microstructure capable of restraining the stress concentration, Hao et al. chose poly (vinyl alcohol) (PVA)/TEMPO oxidized cellulose nanofibers (TOCNF) composites with MXene nanosheets as the sensing layer. They utilized the dynamic interaction between MXene, TOCNF and Fe (II) as the interface crosslinking to alleviate the interlayer stress concentration. The sensor showed competitive advantages of superior thermosensitivity (−1.32%/°C) and outstanding temperature resolution (~0.3  °C) [79], as shown in Figure 8a–c; Phadkule et al. layered the electrospun polyvinylidene fluoride (PVDF) nanocomposite film embedded with silver (Ag) nanoparticles with multi-walled carbon nanotubes (MWCNTs). The sensor showed a negative temperature coefficient (NTC) with an excellent sensitivity of −0.18%/°C and a quick response rate of 11 s and has excellent anti-jamming ability in bending force and wet environment tests [80].

Compared with metallic polymer composites, carbonic conductive polymers have the advantages of simple processing, low cost and high stability. Ryu et al. presented a fiber temperature sensor produced by a thermal drawing process of thermoplastic polylactic acid, reduced graphene oxide and a highly flexible linear low-density polyethylene passivation. The fiber temperature sensor was well-passivated, polymer-nanocomposite-based and exhibited adequate sensitivity (−0.285%/°C) within a temperature range of 25–45 °C and rapid response and recovery times of 11.6 and 14.8 s, respectively [82], as shown in Figure 8d–g; Xiao et al. prepared polyvinyl chloride/carbon black (PVC/CB) temperature-sensitive films by the screen printing method. The prepared sensor exhibited high sensitivity (−0.148%/°C), excellent linearity (R^2^ = 0.995), a fast response time of 0.7 s and good repeatability when used between 18 and 44 °C [83].

Conductive polymers are flexible, wearable, washable and have greater fatigue resistance than metallic materials. Li et al. fabricated a flexible temperature sensor for temperature monitoring by growing a poly (3,4-ethylenedioxythiophene) (PEDOT) thermal layer on the surface of a polyurethane (TPU) fiber. The TPU fiber acted as a strong skeleton, which endowed the composite fiber with good mechanical properties. The obtained sensor has a high sensitivity (0.95%/°C) in the range of 20 °C to 40 °C. The high sensitivity ensured that the sensor still shows a significant temperature response (0.23%) under a 0.2 °C temperature gradient, and its temperature resolution can meet the needs of human body temperature monitoring applications [86]. Fan et al. prepared a thermosensitive fiber coated with polyurethane (PU)/Graphene (3, 4-ethylenedioxthiophene)-poly (styrene sulfonate) (PEDOT: PSS) by combining wet spinning and impregnation in one step. The sensor displayed high sensitivity (−1.72% °C), super resolution (0.1 °C), fast time response (17 s) and high linearity (R^2^ = 0.98) in the range of 30–50 °C [87], as shown in Figure 8h–k.

### 4.4. Flexible Biochemical Sensors

**Figure 9:**
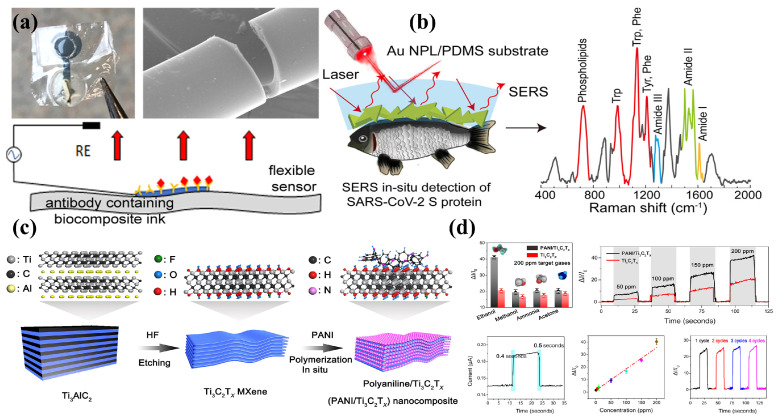
Polymer-based composite conductive materials are used for biochemical sensors. (**a**) Microfabrication of flexible, impedimetric biosensor via photolithography of photo fibroin and sericin-based biocomposites. Reprinted with permission from Ref. [90]. Copyright © 2019, American Chemical Society. (**b**) Au NPLs/PDMS elastomer used as biosensor for the ultrasensitive detection of the SARS-CoV-2 spike protein in cold-chain logistics. Reprinted with permission from Ref. [93]. Copyright © 2019, American Chemical Society. (**c**) Synthesis scheme and characterization of PANI/Ti_3_C_2_T_x_ nanocomposites. (**d**) Sensing performance of PANI/Ti_3_C_2_T_x_-based flexible sensors. Reprinted with permission from Ref. [92]. Copyright © 2019 John Wiley and Sons.

Flexible biochemical sensors show great potential in monitoring human health indicators and environmental changes. For example, they can monitor and analyze chemical substances such as glucose, lactic acid, pH and gas concentration in the environment [88]. Biosensors are usually prepared by selecting a biocompatible polymer that is suitable for soft tissue and combining it with a specific biomarker. Sensitivity, response time, detection range, stability and selectivity are the focus performance of biosensors [89]. Some materials and parameters are partly shown in Table 4. Xu et al. demonstrated a highly sensitive, flexible and labeling free biosensor for detecting vascular endothelial growth factor (VEGF) by combining silk protein with conductive polymer PEDOT:PSS and biometric elements (VEGF antibody). The limits of detection of the sensor to detect VEGF in artificial urine control and urine containing albumin were 22.22 pg·mL^−1^ and 24.87 pg·mL^−1^, respectively (S/N = 3) [90], as shown in Figure 9a. In terms of environmental monitoring, polymer-based conductive composites are used as the sensitive materials of the gas sensor to convert the concentration signal of the target gas into physical signal including voltage, current or resistance [91]. Zhao et al. modified PANI nanoparticles on the surface of Ti_3_C_2_T_x_ nanosheets by in situ polymerization at a low temperature. Using the composite as a sensing material for flexible gas sensors, because of the synergistic properties of composites and the high catalytic/absorptive capacity of Ti_3_C_2_T_x_ MXene, the sensing material displayed both high ethanol sensitivity (41.1%, 200 ppm) and rapid response/recovery time (0.4/0.5 s) at room temperature. These properties render it as a sensing material that can detect real-time VOC gasses effectively [92], as shown in Figure 9d.

### 4.5. Flexible Humidity Sensors

**Figure 10:**
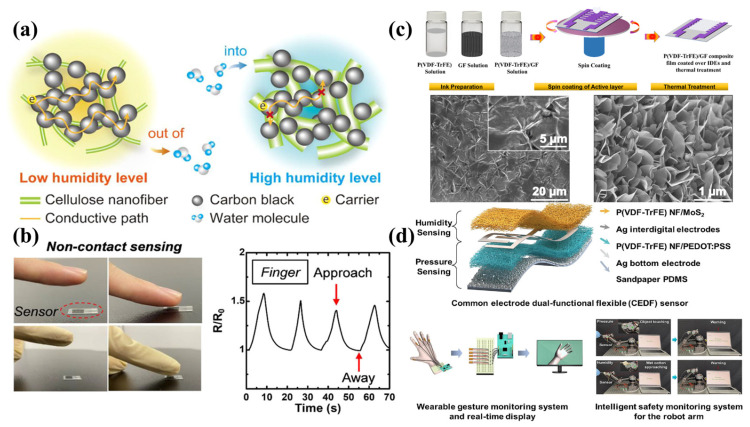
Polymer-based composite conductive materials are used for flexible humidity sensors. (**a**) Schematic of the humidity detection mechanism for the CNF/CB composites. (**b**) Image of a finger approaching the sensor and sensor response. Reprinted with permission from Ref. [100]. Copyright © 2022, American Chemical Society. (**c**) Schematic diagram of the ink preparation of P(VDF-TrFE), graphene flower solution and SEM image of the deposited composite film. Reprinted with permission from Ref. [101]. Copyright © 2021 MDPI. (**d**) P(VDF-TrFE) nanofiber composites for pressure-humidity sensors. Reprinted with permission from Ref. [102]. Copyright © 2023 Elsevier Ltd.

As flexible sensors with non-contact monitoring features, the flexible humidity sensors have shown a variety of applications in human healthcare, plant transpiration and human–computer interaction [97]. The signal strength of a humidity sensor mainly depends on the characteristic of the active material, so the hydrophilicity of the active material plays a crucial role in the sensing performance such as sensitivity, response and recovery time, stability, etc. Some materials and parameters of flexible humidity sensors are shown in Table 5. Flexible humidity sensors based on functional polymers have received extensive research attention due to their biocompatibility and biodegradation characteristics. Polymer-based composite conductive materials used for flexible humidity sensors are shown in Figure 10. Wang et al. prepared PVA/MXene nanofiber film on interfinger electrodes as a humidity-sensitive material using electrospinning technology and fabricated a single-layer MoSe_2_ piezoelectric nanogenerator (PENG) to drive the sensor. The humidity sensor had the characteristics of high response, fast response/recovery time, small hysteresis and good durability [98]; Zhao et al. fabricated a polyaniline/poly(vinylidene fluoride) (PANI/PVDF) flexible membrane through depositing Polyaniline (PANI) on a PVDF microporous membrane. The as-prepared flexible humidity sensor displayed good breathability and the response characteristics of small hysteresis (~5%RH). The PANI/PVDF-integrated flexible humidity sensor could be applied in the non-contact monitoring of respiration and speaking and showed great potential in flexible electronics [99].

## 5. Conclusions and Perspective

The rapid development of integrated, miniaturized and versatile electronics imposes high requirements on flexible sensors. Flexible sensors based on EPCC are playing an increasingly important role in intelligent medical treatment and human–computer interaction. In this review, the material selection, conductive mechanism and flexible sensors based on EPCC are summarized in detail. Although flexible sensors based on EPCC have been extensively studied and considerable progress has been made, there are still many problems to be solved:

1. Stability and flexibility are difficult to combine. The addition of the conductive filler often makes the mechanical properties worse and properties like conductivity and sensitivity become unstable when the sensors are stretched. In addition, the polymer material itself has viscoelasticity. The viscoelasticity will cause the strain to lag behind the change in stress when under a cyclic dynamic load, resulting in hysteresis and poor sensing signal recovery. It is still a challenge to obtain sensors with stable sensing performance and excellent mechanical properties under tensile conditions.
2. Device integration is difficult. The high-density integrated electronic system is realized by stacking multiple layer circuitry through a hole structure to realize interlayer communication. However, the porous structure of the flexible substrate is easy to cause stress concentration, and rupture occurs under tensile deformation. So it is difficult to realize large-area and high-density integrated systems for elastic devices. In addition, the elastic modulus of the materials is different, resulting in stress concentration at the interface in the tensile state, so the interface is easy to damage, which is also a barrier to the integration of flexible electronic devices.

In the field of flexible electronics, properties such as hysteresis, response rate performance, etc., still need to be improved. Sensors based on EPCC need to continuously adapt to human–computer interaction capabilities and intelligence, so that it can be widely used in daily production and life.

## Figures and Tables

**Table 1:** Materials and parameters of flexible pressure sensors.

Materials	Production Methods	Detection Range	Responding Time	Ref.
CNT/PDMS	Mixing	0~350 kPa	0.04 ms	[66]
PDMS/AgNS/RGO	Mixing	0~40 kpa	286 ms	[67]
MXene/AgNWs/TPU	Electrospinning	0~40 kpa	260 ms	[68]
MWCNTs/Graphite/TPU	Mixing/Coating	0~2 MPa	10 ms	[69]
Carbon flower/SEBS	Direct writing printing	0~40 kPa	--	[70]

**Table 3:** Materials and parameters of flexible temperature sensors.

Materials	Production Methods	Detection Range (°C)	Sensitivity (%/°C)	Ref.
MXene/Fe/PTF	Spraying/LBL assembly	20~80	1.32	[79]
MWCNT-Ag-PVDF	Blending/Electrospun	30~40	−0.18	[80]
R-GO/P(VDF-TrFE)	Liquid phase blending	30~80	--	[81]
rGO/PLA	Thermally drawn	25~45	−285	[82]
Carbon black/PVC	Screen-printed	18~44	−0.148	[83]
rGO/CS/PF	Layer-by-layer self-assembly	30~60	−379	[84]
FG/CNT/PDMS	Screen printing	35~85	2.8	[85]
PEDOT/TPU	In situ growing	20~40	0.95	[86]
Graphene/PU/PEDOT:PSS	Wet spinning/Impregnation	30~50°	−1.72	[87]

**Table 4:** Materials and parameters of flexible biochemical sensors.

Materials	Production Methods	Target Substance	Response Time	Sensitivity	Ref.
SPP/PEDOT:PSS/Ab	Spin-coating	VEGF	--	2.62 ± 3.00% (1 pg–10 ng/mL)	[90]
PANI/MXene	In situ polymerization	Ethanol	0.4/0.5 s	41.4% (200 ppm)	[92]
Au NPLs/PDMS	Embed	SARS-CoV-2	--	6.8 × 10^−9^ g/mL	[93]
GG/Ag	In situ synthesis	Ammonia	50 s	~60 (1300 ppm)	[94]
Graphene-PANI	Electrospun	CO_2_	65 s	0.8 (20–100 pm)	[95]
PAni-CoFe_2_O_4_	In situ polymerization	NH_3_	24.3 s	118.3% (50 ppm)	[96]

**Table 5:** Materials and parameters of flexible humidity sensors.

Materials	Production Methods	Humidity	Response	Response/Recovery Time (s)	Ref.
PVA/MXene	Electrospinning	11~52%	40	0.9/6.3	[98]
CNF/CB	Printing	30~90%	120% (ΔR/R_0_)	10/6	[100]
PANI/PVDF	In situ polymerization	11~98%	226.0% (R/R_0_)	1/--	[99]
P(VDF-TrFE/graphene flower	Spin-coating	8~98%	0.0558 pF/RH%	0.8/2.5	[101]
P(VDF-TrFE) NF/MoS_2_	Hydrothermal/hydrothermal	10~90%	230% (ΔC/C_0_)	3.1/5.3	[102]
